# Analysis of RNA expression of normal and cancer tissues reveals high correlation of COP9 gene expression with respiratory chain complex components

**DOI:** 10.1186/s12864-016-3313-y

**Published:** 2016-12-01

**Authors:** Christina A. Wicker, Tadahide Izumi

**Affiliations:** Department of Toxicology and Cancer Biology, University of Kentucky, 1095 V.A. Dr, Lexington, KY 40536 USA

## Abstract

**Background:**

The COP9 signalosome, composed of eight subunits, is implicated in cancer genetics with its deneddylase activity to modulate cellular concentration of oncogenic proteins such as IkB and TGFβ. However, its function in the normal cell physiology remains elusive. Primarily focusing on gene expression data of the normal tissues of the head and neck, the cancer genome atlas (TCGA) database was used to identify groups of genes that were expressed synergistically with the COP9 genes, particularly with the COPS5 (CSN5), which possesses the catalytic activity of COP9.

**Results:**

Expressions of seven of the COP9 genes (COPS2, COPS3, COPS4, COPS5, COPS6, COPS7A, and COPS8) were found to be highly synergistic in the normal tissues. In contrast, the tumor tissues decreased the coordinated expression pattern of COP9 genes. Pathway analysis revealed a high coordination of the expression of the COPS5 and the other COP9 genes with mitochondria-related functional pathways, including genes encoding the respiratory chain complex.

**Conclusions:**

The results indicate that mRNA expression data for the matched normal tissues available in TCGA are statistically reliable, and are highly useful to assess novel associations of genes with functional pathways in normal physiology as well as in the cancer tissues. This study revealed the significant correlation between the expressions of the COP9 genes and those related to the mitochondrial activity.

**Electronic supplementary material:**

The online version of this article (doi:10.1186/s12864-016-3313-y) contains supplementary material, which is available to authorized users.

## Background

The constitutive Photomorphogenesis (CSN) 9, or COP9 signalosome, is a critical multi-functional protein complex in cells [1, 2]. COP9, originally discovered in *Arabidopsis thalania* as a negative regulator of photomorphogenesis, was later found to be highly conserved across many species [3, 4]. COP9 is a 450–550 kDA holocomplex composed of eight subunits whose official gene symbols are listed in Table 1 [1, 5]. COP9 has catalytic activity to remove Nedd8, a small-ubiquitin like modifier, to regulate cullin-RING ubiquitin E3 ligases, and thus protein degradation pathways mediated by cullin complexes [1]. COP9 regulates phosphorylation of proteins such as tumor suppressor p53, and neddylation essential to ubiquitin-mediated proteolysis of key proteins including tumor suppressor HIF-1α [6–10]. Thus, COP9 has been an important focus in cancer genetics [5, 10–13].

**Table 1:** Nomenclature of mammalian COP9 genes

COP9 Genes			
Protein	Gene Symbol	Gene ID	Cytoband
CSN1	GPS1	2873	17q25.3
CSN2	COPS2	9318	15q21.2
CSN3	COPS3	8533	17p11.2
CSN4	COPS4	51138	4q21.22
CSN5	COPS5	10987	8q13.2
CSN6	COPS6	10980	7q22.1
CSN7A	COPS7A	50813	12p13.31
CSN7B	COPS7B	64708	2q37.1
CSN8	COPS8	10920	2q37.3

Despite intense studies in the past, the exact essential role of COP9 still remains unknown. This is largely due to COP9’s promiscuous property to interact with a variety of cullin complexes and others, and to affect stabilities of a number of cellular factors. Additionally, subforms of COP9 complexes were reported [2, 14, 15]. Dubiel et al. proposed that such flexible conformation enables the broad range of substrates.

Among the eight subunits, COPS5 is the most extensively studied subunit that has numerous critical functions including deneddylation reactions [1, 10]. It is commonly over-expressed in cancers leading to increased deneddylation within cells [16]. Other subunits have been shown to possess unique functions. For example, CSN1 (GPS1) is involved in stabilization of p53 [10, 13]. Over-expression of COPS2 is linked to chromosome instability [10, 13]. Both COPS2 and COPS3 knockout are embryonic lethal at E3.5 and E8.5 respectively [10, 13]. COPS6 is frequently over-expressed in cancer [10]. COPS8 knockout is also embryonic lethal (E7.5) with impaired growth and differentiation [10]. Although these reports convincingly indicate that all the COP9 subunits have essential functions in mammalian cells, how these reported functions contribute to COP9 activity remains largely unknown.

Identification of functional groups with which COP9 signalosome may interact in normal physiology may provide an important clue to understand how dysregulation of COP9 may facilitate tumorigenesis.

Deficiency in even one subunit leads to COP9 destabilization. Down-regulation of COPS5 in mouse embryonic fibroblasts drastically decreased the stability of COPS1, COPS3, and COPS8 [17–19]. Therefore, it is plausible that coordinated expression of COP9 genes is needed to maintain necessary amounts of COP9 and imbalance may be associated with disease [20]. To elucidate this possibility, a statistically adequate number of normal tissues must be analyzed with precision for their gene expressions. Using cultured cell lines is not appropriate for this investigation, because the cell lines established in vitro are all transformed and thus expressions of the COP9 genes are likely altered from cells in normal in vivo conditions.

The Cancer Genome Atlas (TCGA) has procured specimens from more than 500 human tumor tissues from various cancers for comprehensive analyses of tumor tissues and advanced cancer genomics. Importantly, matched normal tissues have been analyzed for about 10% of each cancer type. Using the RNAseq expression data in TCGA, we elucidated the possibility that expression of the COP9 genes are synchronized in the normal cells. Indeed the COP9 gene expressions were in synergy in the normal tissues, but this fine coordination was diminished in the tumor tissues. Furthermore, the analysis led to the unexpected observation that the expressions of the COP9 genes were highly synchronized with those involved in mitochondrial biogenesis, particularly those of respiratory chain complex.

## Methods

### Nomenclature of COP9 genes

Although the gene names from CSN1 to CSN8 are intuitive and have been accepted as the names for the COP9 subunits [21], the TCGA uses the official gene symbols. For the simplicity, this study used the official gene symbols as the names of COP9 subunits (Table 1).

### Data obtained from TCGA

The mRNA expression data of normal and tumor tissues were obtained through TCGA’s online data portal site (http://cancergenome.nih.gov). The cancer tissues chosen for this study are 522 cases of head and neck squamous cell carcinoma with 44 normal tissues, as well as lung adenocarcinoma (50 normal and 502 tumor). Normalized gene expression results (mRNASeq2 level3) were collected. Each set of mRNA expression data contained 20,531 gene entries, which were linked to annotations through DAVID functional gene annotation database (Database for Annotation, Visualization and Integrated Discovery; https://david.ncifcrf.gov), yielding 20,154 analyzable gene lists. The RNAseq data were linked to information about the chromosome cytoband and gene ontology (GOTERM_BP_FAT, GOTERM_CC_FAT, GOTERM_MF_FAT, and KEGG_PATHWAY).

Similarly, the de-identified clinical information including anatomical sites and smoking history were retrieved from TCGA. Data processing and analysis were carried out using R (ver 3.1.2) and series of command line programs developed with the Swift language using Xcode ver. 6.

### Generation of correlation coefficient table specific for individual genes

Gene expression data of normal and tumor tissues from donors were assembled into a single table file consisting of columns of tissues and rows of genes. Genes that show zero mRNA values in more than 5% of the tissues analyzed were excluded unless noted otherwise. Pearson’s correlation coefficient (*r*) values between expressions of a target gene (e.g., COPS5) and each of all the other genes were determined, and the genes were then sorted based on the *r* values. Significance of *r* values were assessed with *p* values calculated with cor.test function in R. Pair and boxplots were drawn for particular gene sets using R.

### Expressional correlation of genes closely localized on the chromosomes

#### Generation of cytoband plots

Genes ranked within 500 highest |r| values were pooled, and the cytoband information was processed into float values by the simple formula as following. For a gene with cytoband of ApB.N or AqB.N, a float value was generated by A - (B.N)/100 or A + (B.N)/100, respectively. For example, 8p11.2 was converted to 8–11.2/100 = 7.888, and 8q11.2 was to 8.112. Values of (x, y) = (cytoband, r) was plotted using R. X and Y chromosomes were shown in one column as all analyzed genes mapped in X chromosome were also found in Y chromosome.

#### Consideration of exact transcription start sites

Transcriptional start sites of the COP9 genes were retrieved from the Ensemble database. For a particular geneA in the RNAseq data, a group of genes (Gi) localized within 1 Mbp of the geneA were pooled (the number of genes in Gi = Nh). Then, an enrichment score (ES) for the Gi was calculated based on the correlation coefficient table for geneA [22]. Prior to this calculation, geneA itself (*r* = 1) was removed from the table. Separately, genes localized on the same chromosome but outside of the 1 Mbp of geneA were pooled (Go) and used to calculate control mean ES and standard deviation (sd) for the Nh number of genes that were randomly selected from the Go gene pool. This calculation was repeated for 1000 times to obtain mean and sd values which were used to generate a *p* value based on pnorm function with R. To compare these ES values with other genes, the above calculation was performed with all available genes in the RNAseq data, and the *p*-values were used to calculate false discovery rates (FDR; Benjamini–Hochberg) using p.adjust function in R.

### Interpretation of functional gene annotation

The top 1000 genes with the highest |*r*| values were pooled with each gene accompanying gene ontology (GO) and KEGG pathway information. Over-represented pathways are sorted by the *p* values based on binomial distribution: 1 - pbinom(n, 1000, μ) (in R), where n is the actual count occurring in the 1000 genes, and μ is the average of finding the particular pathways, and calculated with the whole gene set of 20,154.

### Derivation of enrichment score (ES) for KEGG functional pathways

ES scores were calculated by the method described previously [22]. For example, an ES for COPS5 to oxidative phosphorylation pathway was calculated using the COPS5 correlation coefficient table with gene annotations based on the association with “hsa00190” (KEGG i.d. for oxidative phosphorylation) to generate P_hit_ - P_miss_. ES was provided as the maximum value of P_hit_ - P_miss_ as described [22]. A histogram of ES values was calculated with randomly chosen N_h_ genes for iteration of 1000, and the distribution was used to assess the statistical significance.

ESs and corresponding *p* values were calculated for all genes and then false discovery rates were determined as described above.

## Results

### A single gene expression analysis

COPS5 (official gene symbol of CSN5) is responsible for the deneddylation activity of the COP9 signalosome, and also known as Jab1 to activate c-Jun (Jun-activating/binding protein). Thus, most COP9 studies have focused on the activity of COPS5 [23] and its influence on biological activities including tumorigenesis. Because of the pivotal role of COPS5 for the COP9 signalosome in mammalian cells, this study primarily focused on COPS5 to investigate the possibility of coordinated expression of the COP9 subunits in the normal and tumor tissues. Our primary interest was the head and neck squamous cell carcinoma (HNSCC), where effects of smoking and influences of major genetic factors, including p53, p16, AKT1 and K-Ras could be addressed due to its established cancer etiology involving these genes. In addition, HNSCC data in TCGA included a few distinct anatomical sites including oral cavity, tongue and larynx, and thus effects of tissue sites for the gene expression could be elucidated with the clarified clinical record. For the simplicity, the official gene symbols will be used in this study instead of more commonly used protein names (Table 1).

Expression of the COPS5 gene was examined using the data of 44 normal head and neck tissues available in TCGA. COPS5 expression in normal tissues showed mean value of 1261.0 with standard deviation (sd) 328.83 and coefficient of variation (sd/mean) 0.26. As a comparison, mean coefficient of variation of the 15,660 gene set was calculated, and found to be 0.693 (±0.575). Therefore, the COPS5 gene expression appeared to be relatively stable among normal oral tissues.

### Coordinated expression of the COP9 genes in normal tissues

**Fig. 1:**
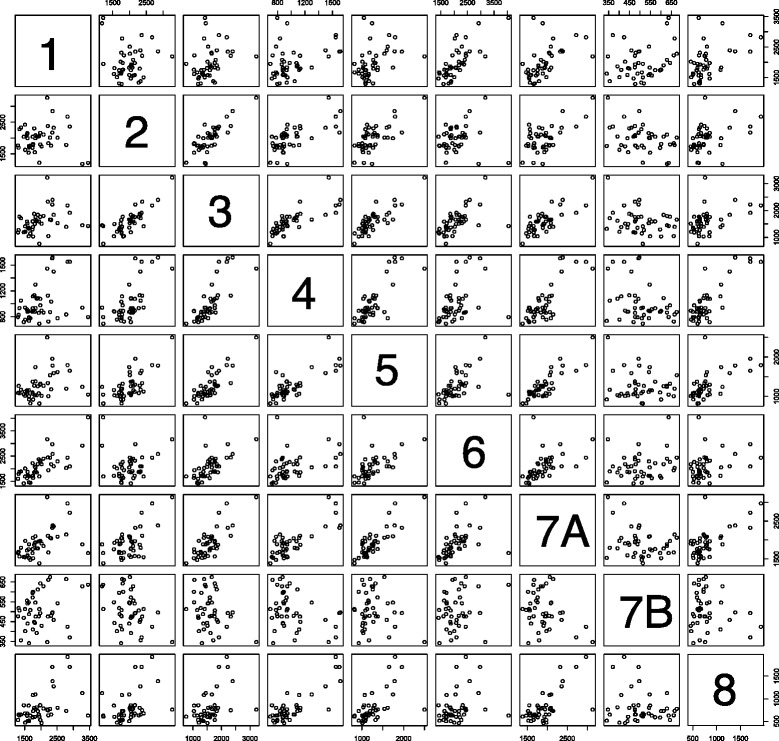
Pair-wise plot of expressions of COP9 genes. The RNA expression results from normal 44 oral tissues are plotted. 1: GPS1, 2: COPS2, 3: COPS3, 4: COPS4, 5: COPS5, 6: COPS6, 7A: COPS7A, 7B: COPS7B, 8: COPS8

Expression patterns of COPS5 and other COP9 genes were elucidated by comparing correlation coefficients (expressed as *r* thereafter), using the RNA expression data (RNAseqV2 level 3). High positive correlations were found among the COP9 gene expressions. Specifically, relative to COPS5, all the other COP9 genes except COPS7B showed statistically significant positive *r* values (Table 2 and Fig. 1). GPS1 (CSN1) showed a smaller *r* value than the others, namely COPS2, COPS3, COPS4, COPS6, COPS7A, and COPS8. The lower correlation of gene expression of GPS1 vs. COPS5 was found to be a general characteristic through our analyses in this study. However, the positive correlation between GPS1 and COPS5 was still statistically significant (*p* = 0.011). In addition to the low *r* value relative to the other COP9 genes, the level of COPS7B expression was significantly lower compared to COPS7A (Additional file 1: Figure S1).

**Table 2:** Correlation of expression of COP9 genes in normal oral tissues

Head and Neck, Normal									
	GPS1	COPS2	COPS3	COPS4	COPS5	COPS6	COPS7A	COPS7B	COPS8
GPS1	1.000	−0.033	0.306	0.369	0.381	0.749	0.514	0.269	0.512
COPS2	−0.033	1.000	0.809	0.687	0.697	0.124	0.613	−0.376	0.411
COPS3	0.306	0.809	1.000	0.776	0.838	0.553	0.785	−0.285	0.455
COPS4	0.369	0.687	0.776	1.000	0.794	0.386	0.805	−0.337	0.831
COPS5	0.381	0.697	0.838	0.794	1.000	0.533	0.851	−0.135	0.619
p	0.01	1.51E-07	1.31E-12	1.28E-10	-	1.98E-04	2.57E-13	0.384	7.69E-06
COPS6	0.749	0.124	0.553	0.386	0.533	1.000	0.495	0.191	0.300
COPS7A	0.514	0.613	0.785	0.805	0.851	0.495	1.000	−0.306	0.673
COPS7B	0.269	−0.376	−0.285	−0.337	−0.135	0.191	−0.306	1.000	−0.161
COPS8	0.512	0.411	0.455	0.831	0.619	0.300	0.673	−0.161	1.000

The RNA expression data obtained from TCGA were analyzed for Pearson’s correlation coefficients. The number of specimens = 44. *P* values for COPS5 vs. other COP9 genes were calculated as described based on paired samples algorithm in R (cor.test (gene A, gene B, method = “pearson”, alternative = “two.sided”)

These results indicated that the expressions of COP9 genes were synchronized in human normal oral tissues. Leppert et al. recently observed that mRNA levels of COP9 genes were regulated by let-7 miRNA [20]. To probe the importance of the regulatory mechanism by miRNA, the TCGA miRNA dataset was used to determine the gene expression correlation of COP9 genes and the let-7 miRNA species (Additional file 2: Table S1). There were statistically significant negative correlations between let-7 isoforms (7a, 7b, 7c, 7e, 7f-2, 7 g) and COP9 genes (GPS1, COPS5, COPS6, and COPS8), which was consistent with the previous work.

### Loss of synergistic expressions of the COP9 genes in cancer tissues

To determine whether the synergistic expressions of the COP9 genes are maintained in the cancer tissues, 42 tumor tissues matched to the normal cases were pooled (2 normal cases could not be matched based on the id tag), and determined their correlation coefficients (Table 3). High positive *r* values were observed among COP9 genes in normal tissues as expected. In contrast, the correlations of expressions among COP9 genes diminished in the matched tumor tissues (Table 3 and Additional file 2: Table S2). Moreover, the negative effect of let-7 miRNA observed in the normal tissue dataset was diminished in the matched tumor tissues (Additional file 2: Table S1). These results demonstrated that the coordinated expression of COP9 genes was diminished in these tumor tissues.

**Table 3:** Loss of coordinated COP9 gene expression in HNSCC

Normal vs. Tumor (paired 42)								
Normal	GPS1	COPS2	COPS3	COPS4	COPS5	COPS6	COPS7A	COPS8
GPS1	1	−0.059	0.303	0.369	0.385	0.75	0.522	0.519
COPS2	−0.059	1	0.783	0.676	0.683	0.072	0.587	0.415
COPS3	0.303	0.783	1	0.775	0.841	0.536	0.777	0.465
COPS4	0.369	0.676	0.775	1	0.786	0.369	0.797	0.835
COPS5	0.385	0.683	0.841	0.786	1	0.521	0.843	0.618
p	0.012	6.25E-07	3.30E-12	6.90E-10	-	4.09E-04	2.37E-12	1.32E-05
COPS6	0.75	0.072	0.536	0.369	0.521	1	0.479	0.296
COPS7A	0.522	0.587	0.777	0.797	0.843	0.479	1	0.675
COPS8	0.519	0.415	0.465	0.835	0.618	0.296	0.675	1
Tumor	GPS1	COPS2	COPS3	COPS4	COPS5	COPS6	COPS7A	COPS8
GPS1	1.000	−0.091	0.055	0.012	0.212	0.360	−0.195	0.218
COPS2	−0.091	1.000	0.126	−0.073	0.218	−0.271	−0.029	−0.066
COPS3	0.055	0.126	1.000	0.043	0.033	0.342	−0.213	0.258
COPS4	0.012	−0.073	0.043	1.000	0.024	−0.023	0.300	0.066
COPS5	0.212	0.218	0.033	0.024	1.000	−0.053	−0.196	0.244
p	0.177	0.165	0.836	0.88	-	0.741	0.213	0.12
COPS6	0.360	−0.271	0.342	−0.023	−0.053	1.000	−0.041	0.205
COPS7A	−0.195	−0.029	−0.213	0.300	−0.196	−0.041	1.000	−0.197
COPS8	0.218	−0.066	0.258	0.066	0.244	0.205	−0.197	1.000

Normal and matched HNSCC tissues (sample number = 42) were compared for their correlation coefficient values among the COP9 genes

### Validation of the results with subgroups (age, anatomical sites, and smoking history)

HNSCC is an age-related disease. The majority of specimens in TCGA are from elderly patients (median = 61; the first quartile = 53, the third quartile = 69), and less than 4% of the patients are younger than 40 years old. Thus, the loss of the coordination of the COP9 gene expressions may be due to the age-related degeneration of the coordination. However, we analyzed the COP9 expression in the 31 tumor tissues that were 42 years old or younger to the 30 tumor tissues that were 80 years old or older (Additional file 2: Table S3 A and B). With this analysis, we found similar loss of the coordinated expressions in specimens from both the young and elderly patients. In addition, there were no significant differences of the expression levels of each COP9 gene between the young and aged groups (Additional file 2: Table S3C). These results support that the age is not a factor causing the low coordination of the COP9 genes in the tumor specimens.

The oral tissues in TCGA were taken from different anatomical sites (oral cavity, tongue, etc.). The synergistic expression of COP9 subunit genes may vary depending on the exact site of oral tissues. To assess this possibility, the data set was arranged into subgroups based on the sites. The major groups were the oral cavity (14 specimens) and the oral tongue (13 specimens). The data sets of the two subgroups were used to determine correlation coefficients of the COP9 genes. These values were similar to those of the entire dataset (Additional file 2: Table S4 and Additional file 2: Table S5), indicating that the synergistic expression of the COP9 genes are maintained regardless of the sites of the oral tissues. Similarly, there appeared to be no clear effects of smoking, because analyzing the normal tissues from eleven current smokers identified a highly similar synergistic RNA expressions of COP9 genes (Additional file 2: Table S6).

### Chromosome mapping of genes of high synergy with the COPS5 expression

**Fig. 2:**
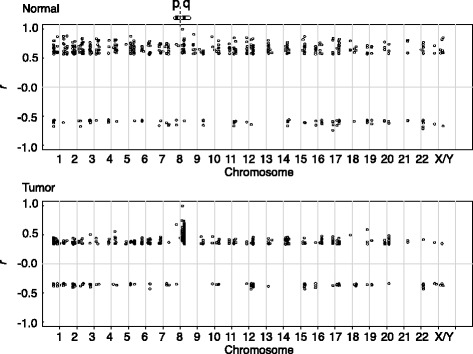
Chromosomal mapping of genes with highly synchronized expression with the COPS5 gene. The 500 highest ranked genes based on |rCOPS5| were pooled, and mapped on to chromosomal locations based on their cytoband information. In the top panel, a cartoon denotes the chromosome 8 (at 8 in x-axis) where the COPS5 gene is located (8q13). The major grid marks on the x-axis (1–22 and X/Y) depict location of centromeres dividing p and q arms at the left and the right sides, respectively. X and Y chromosomes are combined, because all genes analyzed in the figure are mapped in both X and Y

To elucidate the tumor-associated disintegration of the COP9 gene expression in detail, we focused on the COPS5 gene. We generated a “correlation table” of the COPS5, in which expressional correlation coefficients relative to the COPS5 gene (*r*_*COPS5*_) were calculated for all the analyzable genes (about 14,000 genes). By cross-referring the results to gene ontology provided with DAVID, we noticed that genes with high *r*_*COPS5*_ of the tumor tissues were mapped in the same chromosomal location as the COPS5 gene (8q13.2, Table 1). We thus speculated that the loss of the coordination of the COP9 expressions in the tumor tissues was not due to unpredictably disorganized expressions of the COP9 subunit genes, but rather there was a dominant effect of the physical locations of the genes on their expressions. To illustrate this possibility, chromosomal locations of 500 genes with top | *r*_*COPS5*_| values were plotted on chromosomes based on the cytoband information (Fig. 2, top). The genes with high |*r*_COPS5_| in the normal tissues were distributed through all the chromosomes; among the top 100 genes, only seven genes were mapped at 8q, near the COPS5 chromosomal location (8q13.2). In contrast, genes with high |*r*_COPS5_| in the tumor tissues were concentrated near COPS5’s chromosomal region (Fig. 2, bottom); 56 genes out of the top 100 genes were bound to 8q.

We sought to assess the chromosomal cis-effect on the COPS5 gene expression more rigorously. To this end, based on the method by Subramanian et al. we determined the enrichment score (ES) based on the *r*_*cops5*_ for the groups of genes that are localized within 1 mega base pairs (Mbp) from the COPS5, and obtained a *p*-value based on the null hypothesis that these ES values were not different from that calculated for genes outside 1 Mbp from the COPS5 [22]. We have also determined such ES values for all the genes in the chromosome 8 to determine the false discovery rate (FDR, q). The ES value for the COPS5 with the tumor tissues (ES = 0.65, *p* = 0.02, q = 0.043) was significantly higher than that with the normal tissues (ES = 0.39, *p* = 0.27, q = 0.49). The procedure was applied for the other COP9 genes, and revealed significant links to the chromosomal cis-effect in the case of the tumor tissues (Additional file 2: Table S7). Therefore, we conclude that the COP9 expression in the tumors is disintegrated because the chromosomal cis-effect becomes a dominant force to express each subunit gene. Furthermore, these results indicated that analysis of the COP9 expressions in the normal tissues rather than tumor tissues is pivotal for understanding the COP9’s role in vivo, and such an analysis is possible using the RNAseq data in the normal tissues available from TCGA.

### Pathway analysis based on KEGG and GO revealed association of COPS5 expression with mitochondrial pathways

**Fig. 3:**
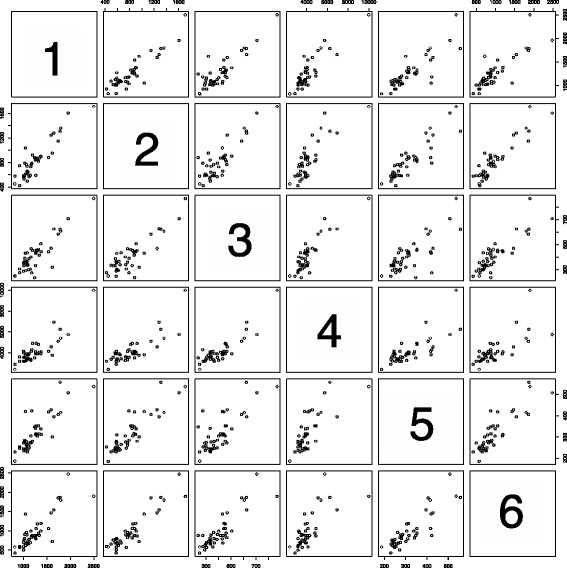
Pair-wise plot for RNA expression 5 mitochondrial genes. The five genes with the highest *r*_COPS5_ in the normal oral tissues are plotted. 1: COPS5, 2: NDUFB6, 3: MRPS36, 4: ATP5F1, 5: TMEM126A, 6: NDUFB3

Genes that belong to particular cell pathways may be over-represented in the COPS5 correlation coefficient table. Identifying such pathways should provide important clues as to COP9’s roles under the normal physiology. Based on the results above, such functional association should be elucidated with the data derived from normal tissues, and the large pool of high quality gene expression data in the TCGA database prompted us to investigate this possibility. Genes with the top 1000 |*r*_COPS5_| values in the normal tissues were pooled, and functional pathways available through GO and KEGG database were associated to each gene (Additional file 3: Table S10). The GO and KEGG pathways were then ranked based on the over-representation, and the 20 most over-represented pathways are shown in Table 4 (the expanded list is shown in Additional file 4: Table S11). Unexpectedly, the pathways related to mitochondrial functions and components were highly over-represented. For example, 61 genes belonging to the oxidative phosphorylation KEGG pathway (hsa00190) were among the genes with high expression correlation with COPS5 out of 106 total genes (*p* < 1.1e-16). A number of GO pathways involving mitochondrial functions were also found among the over-represented pathways. These included respiratory chain (GO:0070469), mitochondrial electron transport (GO:0006120), NADH dehydrogenase activity (GO:0003954), mitochondrial respiratory chain complex I (GO:0005747). In fact, all of the 20 most over-represented pathways are directly related to major mitochondrial and energy derivation functions. At the time this study was carried out, gene ontology database had not yet linked COPS5 to any of mitochondrial-related pathways. Expression values were examined in pair-plots for COPS5 and mitochondrial genes of the five highest *r*_COPS5_, NDUFB6, MRPS36, ATP5F1, TMEM126A, NDUFB3 (Fig. 3). The results illustrate high integrity of the data and the coordination of the expression of the COPS5 with the mitochondria related genes.

**Table 4:** Association of COPS5 with mitochondrial pathways in normal oral tissues

	GO and KEGG pathways	Found	Expected	p	μ
1	GO:0003954 ~ NADH dehydrogenase activity	28	4	1.1E-16	0.00184
2	GO:0006120 ~ mitochondrial electron transport	28	4	1.1E-16	0.00179
3	GO:0070469 ~ respiratory chain	42	6	1.1E-16	0.00323
4	GO:0042773 ~ ATP synthesis coupled electron transport	34	5	1.1E-16	0.00233
5	GO:0042775 ~ mitochondrial ATP synthesis coupled electron transport	34	5	1.1E-16	0.00233
6	GO:0005747 ~ mitochondrial respiratory chain complex I	27	4	1.1E-16	0.00179
7	GO:0030964 ~ NADH dehydrogenase complex	27	4	1.1E-16	0.00179
8	GO:0045271 ~ respiratory chain complex I	27	4	1.1E-16	0.00179
9	GO:0045333 ~ cellular respiration	50	8	1.1E-16	0.00437
10	GO:0044455 ~ mitochondrial membrane part	62	10	1.1E-16	0.00571
11	hsa00190 ~ oxidative phosphorylation	61	10	1.1E-16	0.00576
12	GO:0005746 ~ mitochondrial respiratory chain	36	6	1.1E-16	0.00278
13	GO:0006119 ~ oxidative phosphorylation	48	8	1.1E-16	0.00442
14	GO:0022904 ~ respiratory electron transport chain	35	6	1.1E-16	0.00273
15	GO:0016655 ~ oxidoreductase activity	28	5	1.1E-16	0.00213
16	GO:0022900 ~ electron transport chain	48	9	1.1E-16	0.00516
17	GO:0015980 ~ energy derivation by oxidation of organic compounds	57	11	1.1E-16	0.00670
18	GO:0000313 ~ organellar ribosome	24	5	1.1E-16	0.00238
19	GO:0005761 ~ mitochondrial ribosome	24	5	1.1E-16	0.00238
20	GO:0005743 ~ mitochondrial inner membrane	96	21	1.1E-16	0.01454

Found: Number of genes in a particular GO or KEGG pathway found in the top 1000 *r*_COPS5_ list; Expected: number of expected genes to yield *p* < 0.05 appearing in 1000 genes based on the average appearance per gene (μ); μ values were calculated by the total appearance of individual pathways in the entire gene list of 20,154. *P* values were calculated using binomial distribution in R. Expected occurrences in a list of 1000 genes to provide *p* < 0.05 are listed

Dependency on the anatomical sites and smoking history were tested with the same subgroups as described above (Additional file 5: Table S12). The mRNA expression data from the two major anatomical sites, oral cavity and oral tongue, resulted in almost identical over-representation of the mitochondrial pathways with COPS5. The same conclusion was made with the subgroup containing only the current smokers.

### Alteration of COPS5 expression coordination in cancer tissues

The gene expression coordination between the COPS5 gene and the mitochondrial pathways was elucidated with tumor tissues (Table 5 and Additional file 4: Table S11). Although a few GO pathways related to mitochondrial functions were over-represented, major pathways associated with the COPS5 expression were those involved in ribosome processing and RNA binding as well as membrane lumen pathways.

**Table 5:** Loss of coordinated expression of COPS5 with mitochondria related genes in the tumor tissues

	GO and KEGG	Found	Expected	p	μ
1	GO:0015935 ~ small ribosomal subunit	28	6	1.11E-16	3.08E-03
2	GO:0033279 ~ ribosomal subunit	50	11	1.11E-16	6.30E-03
3	GO:0003735 ~ structural constituent of ribosome	58	13	1.11E-16	7.94E-03
4	GO:0005840 ~ ribosome	68	16	1.11E-16	1.02E-02
5	hsa03010 ~ ribosome	30	8	1.11E-16	4.42E-03
6	GO:0042254 ~ ribosome biogenesis	36	10	1.11E-16	6.10E-03
7	GO:0006414 ~ translational elongation	32	9	1.11E-16	5.06E-03
8	GO:0006412 ~ translation	79	23	1.11E-16	1.60E-02
9	GO:0022613 ~ ribonucleoprotein complex biogenesis	47	14	1.11E-16	9.03E-03
10	GO:0030529 ~ ribonucleoprotein complex	112	34	1.11E-16	2.54E-02
11	GO:0044445 ~ cytosolic part	39	12	1.11E-16	7.49E-03
12	GO:0005739 ~ mitochondrion	154	65	1.11E-16	5.33E-02
13	GO:0044429 ~ mitochondrial part	88	38	1.11E-16	2.90E-02
14	GO:0003723 ~ RNA binding	103	46	1.11E-16	3.61E-02
15	GO:0006396 ~ RNA processing	79	36	1.11E-16	2.75E-02
16	GO:0005730 ~ nucleolus	96	45	1.11E-16	3.47E-02
17	GO:0070013 ~ intracellular organelle lumen	217	104	1.11E-16	8.90E-02
18	GO:0031974 ~ membrane-enclosed lumen	223	108	1.11E-16	9.28E-02
19	GO:0043233 ~ organelle lumen	217	106	1.11E-16	9.10E-02
20	GO:0031981 ~ nuclear lumen	176	86	1.11E-16	7.25E-02

The analysis was carried out in the same way as for Table 5

The possibility that tissues from a particular anatomical site with high dysregulation caused the bias was addressed. Oral cavity, tongue, and larynx are the major three sites that together contribute to about 80% of the donated head and neck cancer tissues. COPS5-associated GO and KEGG pathways were determined for the tissues from the particular sites, and confirmed that correlation of COPS5 expression with mitochondria associated genes were not as predominant as in the normal tissues.

**Fig. 4:**
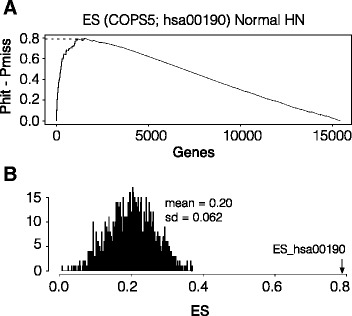
Enrichment score for KEGG oxidative phosphorylation pathway. **a** The enrichment score was calculated for the oxidative phosphorylation KEGG pathway (hsa00190). Genes are aligned on x-axis based on their correlation coefficients to the COPS5 expression. The hsa00190 pathway contained 106 genes in the entire list. **b** 106 randomly selected genes were used to calculate ES using the *r*_COPS5_ gene list as in A. The histogram was generated by iteration of 1000 times. The arrow indicates the ES for hsa00190 (0.797, *p* < 1E-16)

To further support the finding, enrichment score (ES) was calculated for the genes in the oxidative phosphorylation KEGG pathway (hsa00190) using the RNAseq data of 44 normal head and neck tissues [22] (Fig. 4a). The obtained ES value, 0.797, was much higher than the average ES value based on randomly assigned gene pools (Fig. 4b).

In addition, ES values corresponding to all the other KEGG pathways were calculated for the 42 matched normal and tumor tissues (Table 6). The ES value of the oxidative phosphorylation pathway (hsa00190) found to be among the highest. The ubiquinone biosynthesis pathway generated the highest ES value, which is an essential factor in the respiratory chain and requires phenylalanine (generating the second highest ES) for its synthesis. Thus, the enrichment of genes involved in mitochondria-related pathway based on the COPS5 correlation was exceptionally high.

**Table 6:** Enrichment scores for the COPS5 gene on KEGG pathways

	KEGG pathways		ES	Min	Nh	p
1	hsa00130	Ubiquinone and other terpenoid-quinone biosynthesis	0.919	−0.004	6	0.001
2	hsa00400	Phenylalanine	0.811	−0.028	3	0.038
3	hsa00190	Oxidative phosphorylation	0.797	0.000	106	0.000
4	hsa00630	Glyoxylate and dicarboxylate metabolism	0.752	−0.014	13	0.001
5	hsa00053	Ascorbate and aldarate metabolism	0.750	−0.083	6	0.001
6	hsa00062	Fatty acid elongation in mitochondria	0.745	−0.027	8	0.004
7	hsa03050	Proteasome	0.734	−0.004	41	0.000
8	hsa00640	Propanoate metabolism	0.698	−0.003	29	0.000
9	hsa00620	Pyruvate metabolism	0.691	−0.006	34	0.000
10	hsa04260	Cardiac muscle contraction	0.676	−0.001	56	0.000

All KEGG pathways found in the entire gene list of the TCGA RNA expression database (199) were individually scanned through the *r*_COPS5_ alignment, and the maximum (ES) as well as minimum (P_hit_ - P_miss_) were calculated as previously described [1]. The KEGG pathways that generated the 10 highest ES values are shown. N_h_: number of genes belonging to a KEGG pathway in the entire list. A *p* value for a particular KEGG pathway with an N_h_ (= N_k_) was determined using mean and standard deviation obtained by the following method. N_k_ genes were randomly selected from the *r*_COPS5_ list and calculated an ES value, which was reiterated for 1000 times to generate the mean and standard deviation of ES

To elucidate the significance of the COP9 coordination with oxidative phosphorylation compared to other genes, we calculated ES values relative to the hsa00190 KEGG pathway for the entire gene list in the RNAseq data of the matched normal and tumor tissues to calculate FDR (Additional file 2: Table S9). Except for the COPS7B gene, all the COP9 subunit genes showed highly significant correlations with the genes in the oxidative phosphorylation pathway. Not surprisingly, most of the genes in the hsa00190 oxidative phosphorylation pathway resulted in the high ES/low FDR values, validating our approach to detect a linkage of a gene to the oxidative phosphorylation. Remarkably, the COP9 genes (except for COPS7B) were ranked comparably with the hsa00190 genes. The p as well as FDR values were increased for all the COP9 genes (but COPS7B) with the tumors, indicating significant decrease in the association of the COP9 expression to the oxidative phosphorylation related genes. Taken all together, we conclude that there is a high synergy between the expression of the COP9 and mitochondria functions, which implies that the normal physiological function of the COP9 may be inseparable from the mitochondrial activity.

### Analysis of gene expression of other cancers in the TCGA database

Finally, the question was addressed whether the association of the expressions of COP9 genes with those of mitochondrial genes was unique in head and neck tissues. To probe this, the RNA expression data of TCGA for the lung squamous cell carcinoma tissues were analyzed. Tumors originating from the lung upper airway epithelia share similarity with HNSCC in its etiology and its involvement of smoking as an important risk factor. First, the synergistic expression pattern of COP9 genes in the normal tissues (*n* = 50) was examined, and found to be highly synergistic with one another (Table 7 and Additional file 1: Figure S2). Exceptions were GPS1 and COPS7B, which showed lower correlation coefficients to other COP9 factors. The high positive correlations were again lost in the tumor tissues. These results showed a remarkable coincidence with those of head and neck normal and tumor tissues.

**Table 7:** Synchronized expression of the COP9 genes in the matched tissues of normal and lung squamous cell carcinoma

Correlation Coefficient among COP9 genes, Lung Squamous Cell Carcinoma (*n* = 50)									
Normal	GPS1	COPS2	COPS3	COPS4	COPS5	COPS6	COPS7A	COPS7B	COPS8
GPS1	1.000	−0.092	0.268	0.269	0.278	0.717	0.781	0.194	0.244
COPS2	−0.092	1.000	0.792	0.844	0.742	0.300	0.193	−0.432	0.725
COPS3	0.268	0.792	1.000	0.910	0.859	0.557	0.455	−0.373	0.837
COPS4	0.269	0.844	0.910	1.000	0.909	0.603	0.524	−0.279	0.910
COPS5	0.278	**0.742**	**0.859**	**0.909**	1.000	**0.550**	**0.497**	−0.141	**0.859**
p	0.051	6.85E-10	1.33E-15	0.00E + 00	-	3.49E-05	2.40E-04	0.329	1.55E-15
COPS6	0.717	0.300	0.557	0.603	0.550	1.000	0.716	−0.009	0.602
COPS7A	0.781	0.193	0.455	0.524	0.497	0.716	1.000	0.197	0.461
COPS7B	0.194	−0.432	−0.373	−0.279	−0.141	−0.009	0.197	1.000	−0.257
COPS8	0.244	0.725	0.837	0.910	0.859	0.602	0.461	−0.257	1.000
Tumor	GPS1	COPS2	COPS3	COPS4	COPS5	COPS6	COPS7A	COPS7B	COPS8
GPS1	1.000	0.059	0.261	0.228	0.109	0.529	0.307	0.304	0.193
COPS2	0.059	1.000	0.413	0.488	0.142	0.105	0.143	−0.180	0.207
COPS3	0.261	0.413	1.000	0.240	0.267	0.466	0.320	−0.075	0.137
COPS4	0.228	0.488	0.240	1.000	0.235	0.082	0.023	0.076	0.177
COPS5	0.109	0.142	0.267	0.235	1.000	0.233	0.103	0.212	0.170
p	0.452	0.326	0.061	0.101	-	0.103	0.477	0.139	0.239
COPS6	0.529	0.105	0.466	0.082	0.233	1.000	0.640	0.120	0.180
COPS7A	0.307	0.143	0.320	0.023	0.103	0.640	1.000	−0.094	0.008
COPS7B	0.304	−0.180	−0.075	0.076	0.212	0.120	−0.094	1.000	0.613
COPS8	0.193	0.207	0.137	0.177	0.170	0.180	0.008	0.613	1.000

The analysis was carried out in the same way as for Table 3. Values in bold letters indicate significantly high COSP5 correlation with other COP9 components

The functional pathway analysis with the entire gene list resulted in the high correlation of the COPS5 expression with the mitochondrial genes (Additional file 6: Table S13). Unlike the case of head and neck squamous cell carcinoma, however, the coordinated expression of COP9 and the mitochondrial genes was maintained well in the lung squamous cell carcinoma tissues (Additional file 6: Table S13).

## Discussion

Cancer genetics and analyses of the TCGA database mainly focus on the loss of genomic integrity. Mutations and single nucleotide polymorphism in DNA show the high rate of permanent alteration of the genome, epigenetic modification of the genome, RNA and miRNA expression alterations. TCGA data sets are available publicly for these types of analyses. The database is highly reliable and allowed us to examine cancer genomics with more than 500 tumor specimens [24].

In addition to the tumor specimens, data from about 40 to 50 normal tissues are made available for most types of cancers with a notable exception for glioblastoma. Although the data sizes are smaller than those for the cancer tissues, they are arguably one of the most comprehensive databases for genomics of human specimens of non-disease status with pathological and clinical scrutiny, which should provide valuable information for elucidating functions of a gene under the normal physiology. We hypothesized that a synergistic expression of genes may be observed in the normal tissues, when expressions of a group of genes need to be coordinated for their proper cellular activities. COP9 signalosome is an ideal subject to test this possibility, because all eight subunits are essential for the proper COP9 function; deficiency in one of the COP9 genes decreased stability of the other subunits and caused lethality [23, 25]. In this study the COP9 signalosome genes showed highly synergistic expressions in the normal head and neck tissues, except for GPS1 and COPS7B. Remarkably, these characteristics, including the lower r values for GPS1 and COPS7b, were maintained well even in the lung and breast normal tissues (data not shown). The coordinated expression of the COP9 genes decreased in the tumor tissues. Therefore, this coordination could not have been detected by analyzing tumor tissues. Likewise, because all cells cultured in vitro are under non-physiological conditions and thus transformed at some extent, it would have been difficult to obtain convincing evidence for the expression correlation of the COP9 subunit genes based on in vitro cell biological studies alone.

It is interesting to observe the lowest coordination of GPS1 expression compared to the other COP9 components (excluding COPS7B). Besides being a subunit of COP9, GPS1 is known for its function in suppression of G-protein and mitogen-activated signal transduction in mammalian cells [26]. This COP9-independent function quite likely requires a regulation of the GPS1 gene separate from the other COP9 genes.

COPS7B not only had the lowest *r* value, but also significantly lower expression compared to COPS7A (Additional file 1: Figure S1). While COPS7A and COPS7B genes are encoded in different human chromosomes, chromosome 12 and 2 respectively, there is a high homology between COPS7A and COPS7B in the amino acid sequence (78% identical). Thus, COPS7B may have only a minor functional role for COP9, which may be compensated by COPS7A.

To our knowledge, using the exact physical map of the transcription start sites available from the Ensemble genome database has not been applied previously to elucidate effects of the relative locations of genes on the gene expression correlations. In this study, we used this approach to find the chromosomal locations of the genes as a dominant factor causing the loss of the coordinated expressions of the COP9 genes. Much fewer number of genes were under the control of this cis-effect in case of normal tissues, indicating that most genes in normal tissues are regulated independently of their chromosomal locations and perhaps more based on their functional groups, as supported by this study. Therefore, important implications derived from this analysis are that 1) analyzing tumor tissue data may not identify a normal function of a protein family such as COP9, and 2) the qualities of the normal tissue data available in TCGA are high enough to help identifying unknown expressional linkages with functional pathways. Although the primary focus of this study was COP9 expression profiling, we expanded the analysis of the influence of the chromosomal location for all the genes that were analyzable in the RNAseq data, and the data for cancer genes (hsa05200) are shown in Additional file 7: Table S14. It may be interesting to elucidate whether these proto-oncogenes have tight association with the chromosomal locations in other types of tumor tissues available in TCGA.

The observation that the expression of the COPS5 gene (and therefore the other COP9 genes) was synchronized with those involved in mitochondrial function was unexpected. The correlation coefficients of the COPS5 and top ranked genes such as NDUFB6 and MRPS36 (Fig. 3) were remarkably high with statistical significance. Moreover, not only a handful of genes involved in mitochondria but also the total pathway analysis revealed that mitochondria-related cellular functions and pathways were predominantly over-represented in the COPS5 correlation coefficient table. Although there have not been reports that show direct function of COP9 for mitochondria, correlation analyses with the TP53 of which functions have been extensively studied linked it with known cellular functions including cell cycle and DNA damage response pathways (Additional file 8: Table S15). The result validates the approach in this study, and support the prediction that the COP9 signalosome has a novel functional link to mitochondrial activities.

Few studies have linked COP9 to mitochondria-related cellular activities. Jab1/COPS5 was previously identified as an apoptosis inducing factor through modulation of mitochondrial BH3-domain containing protein BclGs [27]. A study found that in *Neurospora crassa*, three subunits of the COP9 were required to recover from deficiency of the alternative oxidase function, which is important for mitochondrial functions [28]. However, no follow up studies have been carried out for mammalian cells. On the other hand, pathways involved in ribosomal functions were associated with the COPS5 expression in the tumor tissues. The significance of this link is currently unclear. However, the COP9 structure is homologous to translation initiation factor 3 (eIF3), another high-ordered complex in the cells [29], and our speculation is that COPS5 may have an overlapping function in the protein synthesis in transformed cells.

The quantity and quality of the genomics database will only be improved. The present study underscores the importance and usefulness of examining the genomics of normal tissues. The results would provide novel functional links of a gene, which was not possible by analyzing the data set of pathophysiological specimens. This approach can reasonably be applied to other genes to identify pathways that have not been known due to scarce information for genomics information of normal tissues.

## Conclusions

The present study investigated COPS5 gene expression using the TCGA database. The analysis revealed the highly synergistic mRNA expression pattern among COP9 genes in normal oral tissues. The coordination was abrogated in the tumor tissues, and the regulatory mechanism was taken over by a *cis*-acting gene regulation. Further analysis revealed an unexpected expression correlation between the COPS5 gene and a number of mitochondria-related genes, postulating the possible functional role of the COP9 signalosome for mitochondrial activities. The quality of the RNA expression data available in TCGA is high enough to allow us gene expression analysis in normal tissues to identify gene functions under normal physiology, which may not be recognized by the data obtained from tumor tissues.

## Additional files

(Additional files Figures and Tables) for Analysis of RNA expression of normal and cancer tissues reveal high correlation of COP9 gene expression with respiratory chain complex components by Christina A. Wicker and Tadahide Izumi. Additional file 1: Figure S1. Expressions of COP9 genes in normal oral tissues. The RNA expression levels (y-axis) of COP9 genes from 44 normal oral tissues are shown. Horizontal lines in the boxes: median values. Top and bottom whiskers denote the first and the third quartile (Q1 and Q3). Outliers (> Q3 + 1.5 * (Q3 - Q1) and < Q1 - 1.5 * (Q3 - Q1)) are shown as individual dots. **Figure S2.** Pair-wise plot for expressions of COP9 genes in the matched lung tissues of normal and squamous cell carcinoma. The plots were generated as described in Fig. 1, except for the data set of the normal (A) and their matched lung squamous cell carcinoma (B) from TCGA was used. The number of samples = 50. (PDF 1508 kb)
Additional file 2: Table S1. The let-7 expression profilesof the matched head and neck normal and tumor tissues. Correlation coefficients for individual let-7 isoforms relative to COP9 genes are shown. Asterisks: *p* < 0.05. **Table S2.** To support the result of Table 3, 2 additional normal head and neck tissues were included to calculate correlation of expressions among the COP9 genes. Correlation coefficients of COP9 genes versus COPS5 (rCOPS5) were determined from randomly selected 44 tumor specimens from the entire pool of 511. The calculations were repeated for 10,000 times to obtain their mean and standard deviation (sd) values, which were used to compare the difference from rCOPS5 of normal tissues. **Table S3.** RNAseq data from young (30 specimens) and elderly patients (31 specimens) were used to calculate the correlation coefficients among the COP9 genes. **Table S4.** 14 normal oral cavity tissues were pooled based on the TCGA clinical data, and the correlation coefficients and their *p* values for COPS5 were calculated as described in the main article. **Table S5.** Data based on the 13 normal tongue tissues were pooled based on the clinical data in the TCGA database, and the correlation coefficients and their *p* values for COPS5 were calculated. **Table S6.** 11 normal tissues from smokers were pooled based on the clinical data in the TCGA database, and the correlation coefficients and their *p* values for COPS5 were calculated. **Table S7.** Nh: number of genes found within 1 Mbp of each COP9 gene; mean, sd, *p* and q values were determined as described in Materials and Methods. **Table S8.** ES for COP9 genes for all KEGG pathways. **Table S9.** ES for COP9 genes for the oxidative phosphorylation pathway. (XLS 80 kb)
Additional file 3: Table S10. The COPS5 correlation coefficient table list includes 1000 highest |*r*| values relative to the expression of COPS5 in the normal and tumor tissues of head and neck. (XLSX 621 kb)
Additional file 4: Table S11. GO and KEGG pathways that are over-represented in the normal and tumor oral tissues based on the COPS5 correlation coefficient table. (XLSX 147 kb)
Additional file 5: Table S12. GO and KEGG pathways over-represented by COPS5 expression correlation in the two major anatomical sites in the head and neck (oral cavity and tongue). (XLSX 57 kb)
Additional file 6: Table S13. GO and KEGG pathways over-represented in the normal and tumor lung tissues based on the COPS5 correlation coefficient table. (XLSX 44 kb)
Additional file 7: Table S14. Genes under the chromosomal cis-effect in the HNSCC tissues (XLS 2117 kb)
Additional file 8: Table S15. KEGG pathway analysis for the TP53 gene (XLS 70 kb)
