# Supplementary figures and images for: Analysis of RNA expression of normal and cancer tissues reveals high correlation of COP9 gene expression with respiratory chain complex components
Source: BMC Genomics. 2016 Dec 1;17:983. doi: 10.1186/s12864-016-3313-y (PMC5131501; doi:10.1186/s12864-016-3313-y)

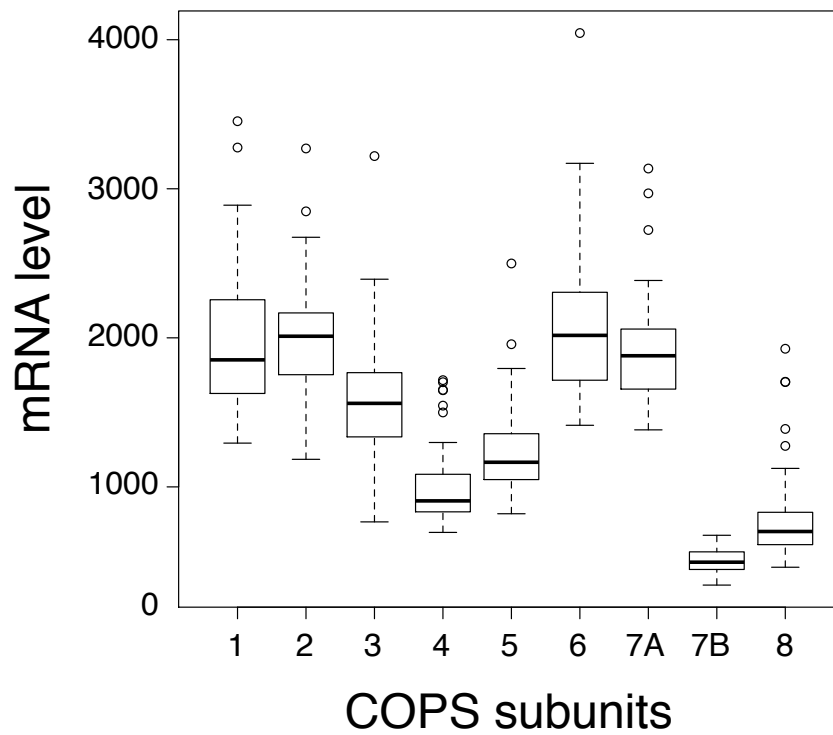

Fig. S1

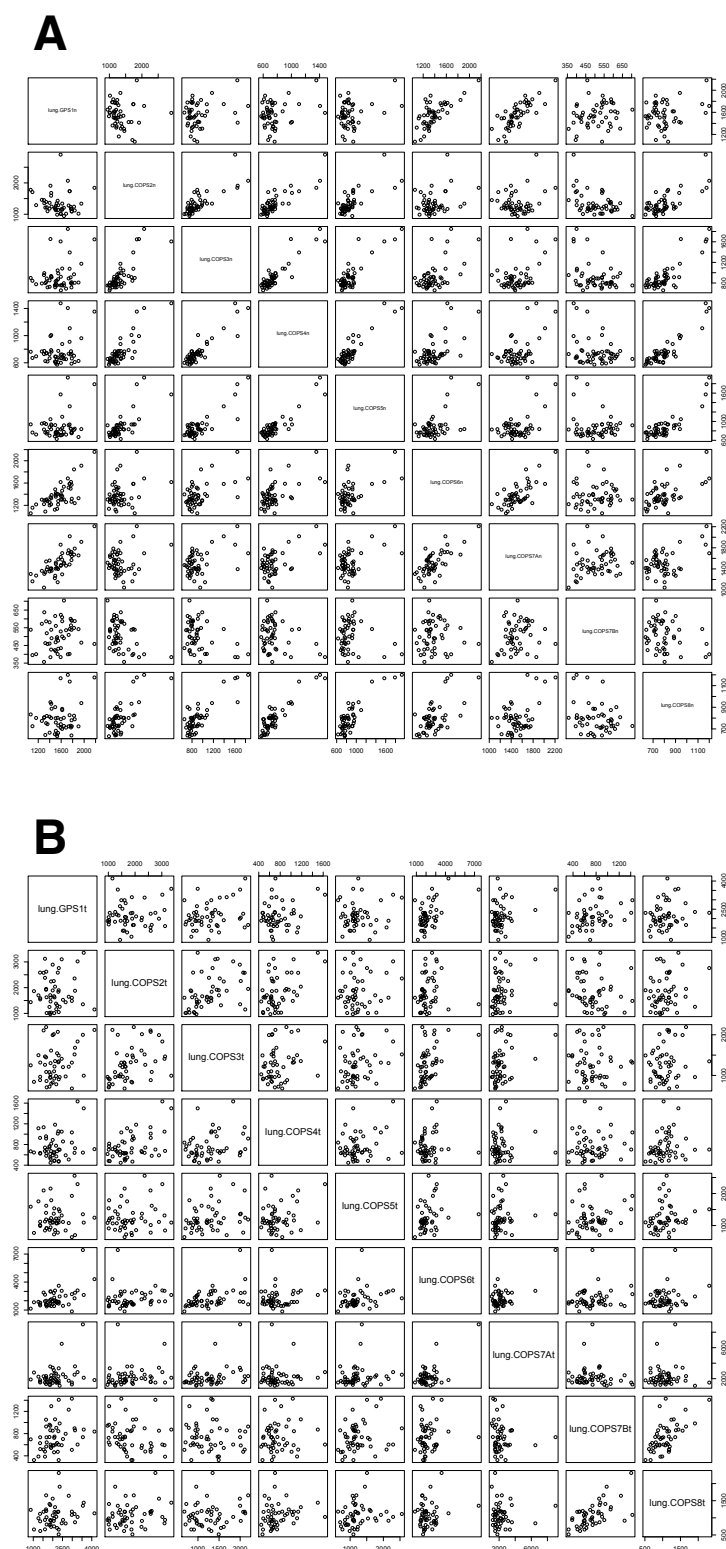

**Fig. S2**

Wicker and Izumi

Supplement: Additional file 1: Figure S1. — Expressions of COP9 genes in normal oral tissues. The RNA expression levels (y-axis) of COP9 genes from 44 normal oral tissues are shown. Horizontal lines in the boxes: median values. Top and bottom whiskers denote the first and the third quartile (Q1 and Q3). Outliers (> Q3 + 1.5 * (Q3 - Q1) and < Q1 - 1.5 * (Q3 - Q1)) are shown as individual dots. Figure S2. Pair-wise plot for expressions of COP9 genes in the matched lung tissues of normal and squamous cell carcinoma. The plots were generated as described in Fig. 1, except for the data set of the normal (A) and their matched lung squamous cell carcinoma (B) from TCGA was used. The number of samples = 50. (PDF 1508 kb) [file 12864_2016_3313_MOESM1_ESM.pdf]
